# Aptamer-Enabled Nanomaterials for Therapeutics, Drug Targeting and Imaging

**DOI:** 10.3390/cells11010159

**Published:** 2022-01-04

**Authors:** Mengping Liu, Lin Wang, Young Lo, Simon Chi-Chin Shiu, Andrew B. Kinghorn, Julian A. Tanner

**Affiliations:** 1 School of Biomedical Sciences, Li Ka Shing Faculty of Medicine, The University of Hong Kong, Hong Kong SAR 999077, China; liump2017@gmail.com (M.L.); wanglin9@connect.hku.hk (L.W.); young.lo@alumni.ubc.ca (Y.L.); simon.chichin.shiu@gmail.com (S.C.-C.S.); andrew.b.kinghorn@gmail.com (A.B.K.); 2 Advanced Biomedical Instrumentation Centre, Hong Kong Science Park, Shatin, New Territories, Hong Kong SAR 999077, China

**Keywords:** nanomaterials, aptamers, therapy, drug delivery, bioimaging

## Abstract

A wide variety of nanomaterials have emerged in recent years with advantageous properties for a plethora of therapeutic and diagnostic applications. Such applications include drug delivery, imaging, anti-cancer therapy and radiotherapy. There is a critical need for further components which can facilitate therapeutic targeting, augment their physicochemical properties, or broaden their theranostic applications. Aptamers are single-stranded nucleic acids which have been selected or evolved to bind specifically to molecules, surfaces, or cells. Aptamers can also act as direct biologic therapeutics, or in imaging and diagnostics. There is a rich field of discovery at the interdisciplinary interface between nanomaterials and aptamer science that has significant potential across biomedicine. Herein, we review recent progress in aptamer-enabled materials and discuss pending challenges for their future biomedical application.

## 1. Introduction

**Figure 1:**
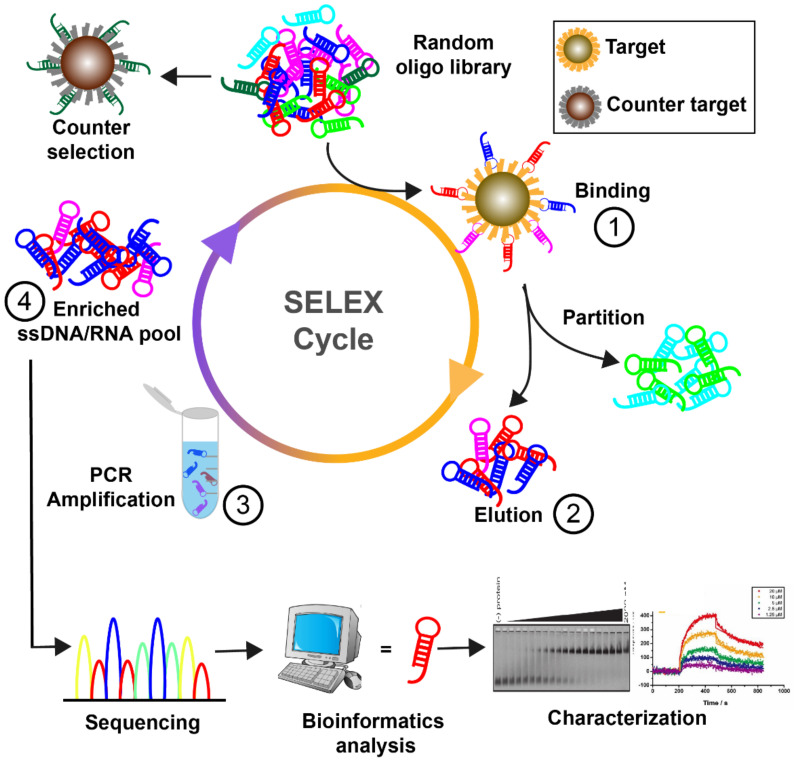
Illustration of the aptamer selection process. Typically, four steps are involved in the SELEX cycle. Step 1, the single-stranded DNA or RNA library is incubated with target molecules. Step 2, the bound sequences are separated from unbound strands and recovered for further process. Step 3, the target-binding sequences are amplified by PCR. RNA molecules need additional transcription procedures for amplification purposes. Step 4, single-stranded DNA/RNA sequences are re-generated from PCR products as a new library for the next round of selection. Through several iterative cycles, aptamers can be identified by sequencing and characterization assays.

Nucleic acid aptamers emerged in the 1990s as functional single-stranded DNA or RNA molecules exhibiting binding properties, typically in the range of 20–80 nucleotides [1]. Aptamers are generated via a process called systematic evolution of ligands by exponential enrichment (SELEX). They fold into unique three-dimensional structures to achieve specific recognition of various targets including small chemical molecules, proteins, genes, metal ions, whole cells or even tissues. (Figure 1) [2]. Thus, aptamers have been termed as “chemical antibodies” which can offer advantages over protein-derived antibodies including simple synthesis, flexible structure, ease of quality-controlled production, and feasible access to small molecules or hidden domains within protein targets [3]. For therapeutic applications, aptamers can serve as the direct molecules of therapeutic action or drug carriers [4]. Although extensive proof of concept studies have shown promise for aptamer-based treatments, there has been only one RNA aptamer named as pegaptanib (Macugen; Pfizer/Eyetech, New York, NY, USA) approved for medical practice [5]. In comparison with antibodies, the clinical translation of aptamers is less developed, for reasons including inherent physicochemical limitations such as nuclease susceptibility, renal filtration and challenges in the physiological environment [6]. Nevertheless, in recent years, rapid advances especially in material science are providing new opportunities for aptamers in biomedical application.

**Figure 2:**
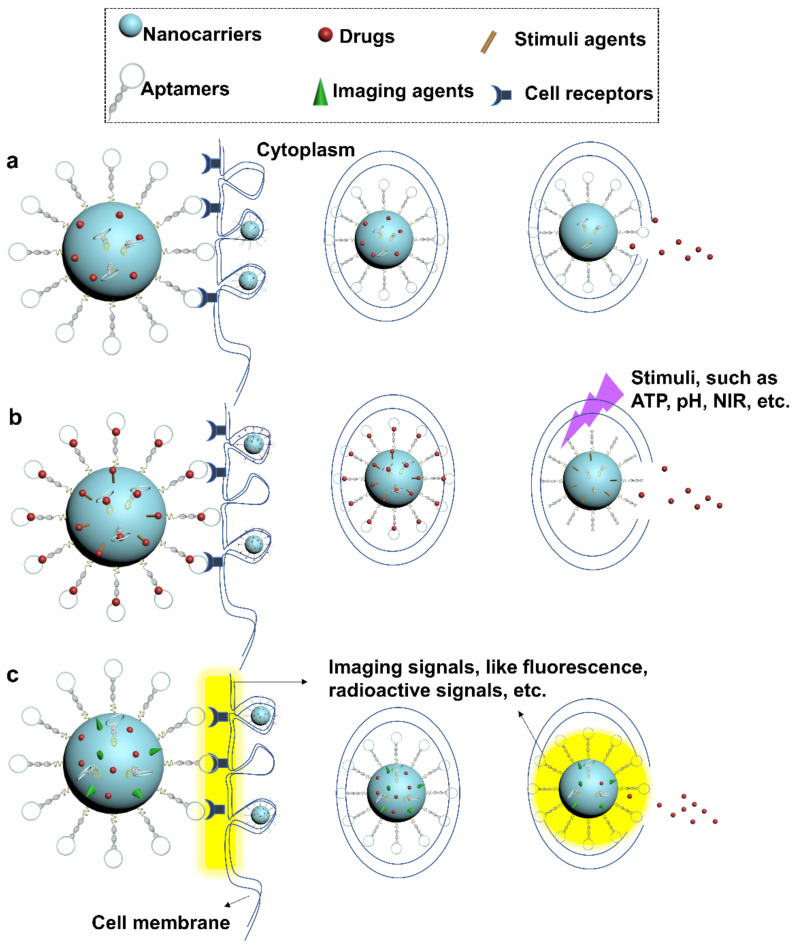
Applications of aptamer-functionalized drug delivery nanocarriers. Aptamer-incorporated drug nanocarriers can be designed for (**a**) targeted drug delivery, (**b**) controllable drug release, and (**c**) imaging-guided therapy. In (**b**), drugs captured by conformation-switchable aptamers or other stimulus-responsive agents can be programmatically released in response to the environmental stimuli. In (**c**), imaging signals can be designed to release with payloads or upon binding to target cells, enabling guiding and tracking of therapeutics in vivo.

A variety of natural or synthetic nanomaterials have been developed for biomedical application including DNA nanostructures [7], polymeric micelles [8], silica nanoparticles [9], gold nanoparticles or carbon nanotubes [10,11]. They are commonly used to develop favorable formulations for different therapeutic agents, particularly insoluble compounds or biotherapeutics [12,13]. The properties of biocompatibility, biodegradability, large loading capacity, and enhanced permeability and retention (EPR) effect, benefit such materials-based formulations. This can lead to prolonged circulation time, increased cellular uptake, and enhanced therapeutic efficacy [14]. However, most drug delivery materials lack specificity towards pathological targets, resulting in limited therapeutic effects and systematic cytotoxicity [15]. Therefore, functionalizing drug carriers with targeting moieties has become a focus of research. Aptamers have remarkable targeting specificities, so can benefit traditional nanocarriers through targeted delivery, increased drug permeability, reduced untargeted cytotoxicity, improved drug efficacy, and controllable drug capture and release [15,16]. Due to the large surface area, or EPR effects, some delivery materials in turn are able to enhance the functions of aptamers and protect them from nuclease degradation and quick filtration [17]. Moreover, the ease of introducing modifications on aptamers or nanomaterials, has further allowed researchers to design versatile drug delivery systems. Additive functions such as stimuli-responsive drug release and real-time imaging have been achieved in some aptamers-based drug carriers (Figure 2) [18,19]. Such progress holds promise for aptamer-functionalized materials for extensive therapeutic applications.

In this review, we frame the current development and applications of several aptamer-conjugated nanomaterial therapeutic systems. Progress and challenges in clinical translation will also be discussed, leading to a view of their potential for practical applications.

## 2. Aptamer-Functionalized Biological Materials for Therapeutic Applications

The generalized concept of biological materials is biocompatible materials that originate from natural living structures that perform, augment, or replace a natural function. Biological materials are commonly designed and engineered for medical and pharmaceutical purposes from a precise nanotechnology perspective. Aptamers are capable of enriching the bioactivity of biological materials due to their programmability and compatibility. To date, aptamers have been incorporated into different biological materials including antibodies, RNAi reagents, DNA origami, and microsomes, to enhance their activities and functions.

### 2.1. Biologics

Biologics are products that are derived from or contain living organisms. Such biological materials are important for advanced therapy of a variety of human diseases due to their extensive therapeutic functions and relatively low side effects when compared to chemicals. Proteins and nucleic acids are mainstream biologics. They present impressive efficiency in treating diverse human diseases such as cancers [20], rheumatoid arthritis [21], and diabetes [22]. However, more widespread application remains hindered by limited cellular uptake or nonspecific targeting. Aptamers, as antibody-mimic materials, have been regarded as a “key” to resolve those problems due to their remarkable targeting abilities. Below we will review recent progress of using aptamers to empower different biologics.

#### 2.1.1. Protein Drugs

Short peptides and antibodies have been developed for combatting various diseases [23]. However, the use of protein drugs has been limited by constraints such as lack of cell-specific targeting ability and limited tumor penetration [24,25]. Aptamers can show cell-type specificity, efficient tumor penetration, and low immunogenicity, presenting as strong candidates to amplify the advantages of protein drugs [3,26].

Aptamer-antibody composites show particular promise. Aptamers and antibodies are able to mutually benefit each other to maximize their therapeutic efficiency. In the study of Kyun Heo et al., authors developed an aptamer-antibody complex, named “oligobody” for targeted cancer therapy [27]. They conjugated cotinine with t44-OME, an aptamer against vascular endothelial growth factor (VEGF), and an anti-cotinine antibody. Results showed that the aptamer-antibody complex could penetrate deeply into tumor tissue, whereas the anti-VEGF antibody could not. On the contrary, the antibody part in the complex significantly improved the pharmacokinetics of the aptamer and did not affect its affinity. Similarly, Passariello M. et al. developed a novel bispecific aptamer-antibody conjugation, by linking anti-epidermal growth factor receptor (EGFR) aptamer with an anti-epidermal growth factor receptor 2 (ErbB2) compact antibody or with an immunomodulatory (anti-PD-L1) antibody [28]. The aptamer-antibody conjugation enhanced the cancer cell killing potency and redirected and activated the T cells against cancer cells. They proved that aptamer-antibody conjugation not only increased cell-type specificity, but also improved pharmacokinetic and pharmacodynamic properties of aptamers due to combined advantages from antibody and aptamers [28]. Apart from antibodies, aptamers can also be conjugated with short peptides to enhance their bioactivity and specificity. Rajabnejad et al. generated an aptamer-peptide complex by conjugating an anti-nucleolin aptamer (AS1411) and metilin to target cancer cells [29]. Metilin is a 26-amino-acid long peptide with known anticancer properties by inducing cell lysis [30]. Although, many studies have reported metilin inhibits cancer cell growth, it can also induce serious side effects including liver injury and haemolysis [30]. By conjugating with 5’ end NH2-modified AS1411, Metilin-AS1411 specifically targeted A549 (nucleolin positive) cell line, while was poorly delivered into L929 (nucleolin negative) cell line. In addition, Metilin-AS1411 complex inhibited the haemolysis activity of single metilin in human blood.

Aptamers also can enhance the bioactivity of protein scaffolds for different applications. Anusha Pusuluri et al. developed an aptamer-peptide synergistic drug conjugate to combine chemotherapy drugs at specific molar ratios to achieve a higher potency [31]. Researchers conjugated the anti-nucleolin aptamer to a drug-preloaded peptide, thereby delivering the chemotherapy drugs doxorubicin (DOX) and camptothecin (CPT) at an optimal ratio. Normally, combined chemotherapy drugs are administered at maximum tolerated dose to avoid sub-optimal exposure to individual drug. However, this approach may also introduce many side effects and may diminish the therapeutic drugs. By using the aptamer-peptide synergistic drug conjugation, these limitations can be prevented. This design achieved anti-tumor efficacy in vivo at an extreme low concentration of DOX (500 µg/kg a dose) and CPT (350 µg/kg a dose), which are 20–30-fold lower than their reported maximum tolerated dose. Inspired by the Large Latent Complex (LLC), Anna Stejskalová et al. designed an aptamer-protein scaffold for cell-selective release of growth factors [32]. They developed Traction-Force Activated payloads (TrAPs), consisting of a growth factor aptamer in which one end was attached to an integrin binding peptide and another end was chemically modified for nonspecific conjugation with cellular substrate and scaffolds. Upon the traction forces induced by integrin expressing cells, the aptamer structure was unfolded thereby releasing the bound growth factors.

#### 2.1.2. Nucleic Acid Drugs

Nucleic acid drugs have been used for a long time to combat various diseases since RNA interference (RNAi) was found in 1990 [33]. Nowadays, the toolbox of Nucleic acid drugs has expanded extensively, including siRNA, antisense nucleotide, miRNA, mRNA and aptamers [34,35]. A variety of delivery platforms have been developed to deliver RNA nucleotide drugs. Of these, viral vectors are commonly used but are constrained by immunogenicity, mutagenesis, and biohazards [36]. Non-viral vectors such as liposomes are safer but liposome-mediated transfections are non-specific, leading to limited drug efficacy [37,38]. Therefore, a more specific and biologically safer delivery approach for nucleotide drugs is needed.

Aptamers as a carrier system for siRNA drugs have been extensively explored during the last decade. Aptamers could be conjugated with siRNA therapeutic drugs in a covalent and noncovalent ways. Aptamer-mediated delivery of siRNA was first introduced by two independent groups in 2006 [39,40]. Since the siRNA and aptamers are all nucleic acids, the aptamer-siRNA conjugates (AsiCs) could be obtained via straightforward covalent linkage or complementary annealing. In addition to covalent conjugation, a ‘U-U-U’ linker was introduced to connect the aptamer and siRNA for delivery of siRNA into aptamer-targeting cells [41]. For non-covalent conjugation, a streptavidin connector was used to connect two biotinylated aptamers and siRNA and this study showed RNAi activity in aptamer targeting cells [39]. Zhou et al. proposed a universal “sticky bridge” to facilitate attachment of siRNA onto chemically synthesized aptamers via complementary annealing [42]. Their aptamer-siRNA chimera successfully inhibited HIV-1 replication and infectivity in cultured T cells and primary blood mononuclear cells. For more detail, the conjugation strategy and applications of aptamer-siRNA conjugates have been well reviewed by others [39,43,44].

More recently, Jeong et al. introduced a multivalent aptamer-siRNA conjugates for delivery of DOX/siRNA into drug-resistant cancer cells [45]. Multivalent aptamer-siRNA conjugates contained aptamers against mucin-1 and siRNA against bcl-2 genes. The DOX was loaded into the conjugates by intercalation with nucleic acids. This multivalent aptamer-siRNA conjugates provide a system for combining chemotherapeutics and siRNA drugs to overcome the multidrug resistance in many cancer cells. Similar to aptamer-siRNA conjugates, aptamer was also used to mediate the delivery of therapeutic aptamers. Li et al. introduced a novel bifunctional aptamer for blood-brain barrier and tauopathy therapy [46]. In this study, they synthesized an aptamer containing one transferrin aptamer to facilitate transcytosis across blood-brain barrier endothelial cells and one Tau protein aptamer to inhibit Tau phosphorylation in the brain. Two aptamers with complementary overhang formed a circular bifunctional aptamer through hybridization and T4 enzymatic ligase. Such circular bifunctional Tau aptamers extended the serum stability and exposure to the brain, as well as improved memory in mouse models.

### 2.2. DNA Nanostructures

DNA is the fundamental genetic material. The field has moved forward significantly since the discovery of the crystal structure of double helix by James Watson and Francis Crick in 1953 [47]. Generally, genomic DNA is topologically linear, while DNA can also exist in various conformations. Cytosine-rich i-motif and guanine-rich quadruplex have been discovered in telomeres as a cause of cancer development and tumor suppression [48,49,50]. Nonetheless, there are branched DNA as intermediates during DNA replication, recombination, and repair. The branched DNA structures, such as the Holliday junction, inspired Nadrian Seeman to propose DNA as chemical materials for assembly of nanoscale molecular structures in 1982 [51,52]. The programmability and specificity of Watson-Crick base pairing enable design and assembly at the nanometer scale [53]. There mainly are two ways to assemble DNA nanostructures. One is using short synthetic DNA strands to form “DNA tiles”, which mirror the principle of Lego bricks. The second fabrication process is usually called DNA “Origami”, which is a Japanese word that means paper folding. DNA origami is a bottom-up assemble process, which uses hundreds of short staple strands to fold a single-stranded scaffold strand of a few thousand nucleotides into a well-defined object [54,55,56,57,58] The biocompatibility and low immunogenicity of DNA make it ideal for biological applications. Therefore, DNA nanotechnology holds significant potential for the development of “smart” drug delivery systems due to its programmable structure to facilitate drug loading and cellular delivery. The three-dimensional shape and size of DNA nanostructure is highly controllable and can be maintained around 50 nm diameter as the optimal size for cell entry [59,60,61,62,63].

**Figure 3:**
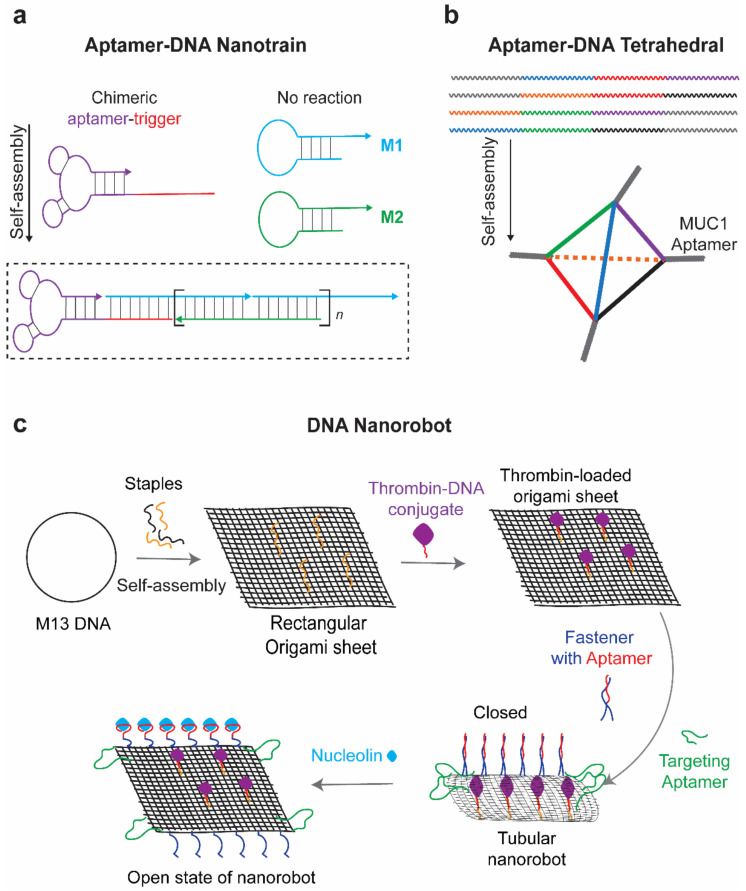
Schematic representation of DNA nanostructure assembly. (**a**) DNA nanotrain with aptamers assembled via complementary sequence. Modified with permission from reference [64]. Copyright 2013 PNAS. (**b**) Assembly of DNA tetrahedron through simple extension of strands. Modified with permission from reference [65]. Copyright 2019 RSC publishing. (**c**) Aptamer guided and gated the delivery of therapeutic drugs. Adapted from reference [66]. Copyright 2017 ACS publications.

Despite promising delivery capacity, DNA nanostructures still lack the targeting ability to deliver drugs in a specific manner. Incorporation of aptamers onto DNA nanostructures can endow them with cell targeting ability for drug delivery. DNA nanostructures and aptamers all originate from nucleic acid materials. Aptamers can be easily assembled onto DNA nanostructures. For example, Tan’s group reported a DNA nanotrain, where an aptamer was assembled on the DNA nanostructure through a complementary sequence (Figure 3a) [64]. Other groups decorated the DNA tetrahedron with anti-cancer aptamers via simple extension of aptamer sequence at end of each tetrahedron DNA strands (Figure 3b) [65,66].

In clinical application, the side effect of chemotherapy drugs such as doxorubicin (DOX) significantly harms the life quality of cancer patients. DOX has binding affinity to DNA duplexes to form a stable complex, so encapsulation of DOX by DNA nanostructures may provide a novel drug delivery system for chemotherapeutics Therefore, aptamer-functionalized DNA nanostructures have been used to deliver the DOX in a precise manner, reducing the side effects of the drug in non-tumor cells and tissues [67,68,69]. Apart from DNA binding small molecules, RNA drugs can also be delivered by DNA nanostructures to overcome degradation by serum nuclease. Lv et al. reported a self-assembled DNA dendritic nanostructure for gene silencing. The outer layer of dendrimers has two overhangs to hybridize with sgc8c aptamers (specific to PTK-7-overexpressed CEM cells) and siRNA, resulting in a better siRNA delivery system with stronger targeting ability and less cytotoxicity compared with lipofectamine [70].

Apart from targeting efficacy, aptamers also help to gate the release of drugs encapsulated in the DNA nanostructure. With the help of aptamers, the strand-displacement principle can be used to expose the internal cavity of three-dimensional DNA origami and release the drugs [71]. The aptamer-gated DNA origami was firstly designed by George Church and was then applied for encapsulation and targeted delivery of thrombin to tumor blood vessels. The DNA origami nanorobot was functionalized with a nucleolin aptamer delivered thrombin to blood vessels in targeted tumors (Figure 3c) [72]. The DNA nanorobots were functionalized with nucleolin targeting Aptamer on the surface and conjugated with blood coagulation protease thrombin within its inner cavity. Anti-nucleolin aptamers on the nanorobot not only guided the targeted delivery, but also serve as a molecular trigger to unlock the nanostructure and expose the thrombin in cancer blood vessels. This is a precise drug delivery platform and presents promising cancer therapeutics in mouse models. Additionally, immune responses induced after origami treatment are low. Similarly, a DNA dendritic nanostructure composed of three aptamers was designed for targeted and controlled delivery of anti-tumor drug epirubicin to cancer cells [73]. Anti-cancer aptamers Mucin 1 (MUC1) and AS1411 were conjugated onto the surface of the DNA dendrimers to assist the targeted delivery. Meanwhile, ATP aptamers within the DNA dendritic nanostructure facilitate the disassembly of dendrimers in ATP-enriched lysosomes, leading to the release of carried drugs. In addition, our group developed an aptamer-gated DNA nanobox, which could sense the malaria diagnostic protein *Plasmodium falciparum* lactate dehydrogenase (PfLDH) to control the box opening [74]. This aptamer-gated platform was characterized by transmission electron microscope and holds a potential for targeted drug delivery. Additionally, DNA nanostructures can also collaborate with other biological materials. For example, DNA nanostructures are also used as backbone materials for hydrogel assembly [75,76]. By incorporating with aptamers, a dynamic and bio-responsive hydrogel can be developed based on aptamer-target interaction [77,78]. An aptamer strand, capturing the specific target, was used to initiate the crosslinking of DNA hydrogel and gate the release of captured drugs. DNA strands complementary to the aptamer could trigger the release of potential therapeutic drugs including thrombin and adenosine [79].

### 2.3. Other Biological Materials

The CRISPR/Cas9 system is now well established for laboratory genome editing due to its high specificity and other advantages. Further development of CRISPR systems will require delivery to specific cells and active sites. By a post insertion approach, Zhen et al. developed an aptamer-functionalized-liposome-CRISPR/Cas9 against Polo-like kinase 1 in prostate tumor cells [80]. Similarly, Liang et al. selected an osteosarcoma cells specific aptamer and conjugated it with PEG-PEI-Cholesterol (PPC) lipopolymer containing plasmids coding for CRISPR/Cas9 and VEGFA gRNA sequences [81]. They showed gene editing and tumor suppression in a cell-specific manner. In addition, researchers in these two reports demonstrated the efficacy of aptamer functionalized CRISPR/Cas9 in animal models.

Although many synthetic delivery platforms are promising, potential toxicity and immunogenicity and low specificity halted the translation from bench to bedside. Exosomes are naturally present nanoparticles in the human and animal body, so the high biocompatibility and low immunogenicity make exosomes as an ideal candidate for drug delivery. Exosomes are nanoscale membrane vesicles (30–100 nm in diameter), which were initially described as micro-vesicles secreted from neoplastic cells [82,83]. The natural nanoparticles had long been viewed as waste from cells until Lötvall et al. showed that some exosomes transfer mRNA and microRNA to make proteins and regulate gene expression [84]. In 2017, an American research group used exosomes to delivery siRNAs against mutated Kras in pancreatic cancer, which was a major challenge for conventional RNAi delivery systems, such as liposomes [85]. They also pointed out that “don’t eat me” signal, CD47, on the surface of exosomes enhances retention of exosome through protection from phagocytosis [85]. Meanwhile, aptamers were also used to develop a targeted exosome delivery system. Zou et al. developed a diacyllipid-aptamer conjugation to functionalize the exosome for cancer treatment [86]. They conjugated the diacyllipid with aptamer sgc8 (specific to CCRF-CEM cells) through a PEG linker, then diacyllipid-aptamer conjugation was decorated onto the exosomes collected from immature dendritic cells. By combining the target capacity of aptamer and advantages of natural exosome vesicles, sgc8-aptamer-functionalized exosomes specifically delivered DOX to cancer cells. Similarly, Fazileh et al. developed an aptamer-functionalized exosome by covalent conjugation of carboxylic functionalized-Aptamer onto amine groups on exosome membranes [87]. LJM-3064 aptamer, recognizing myelin and inducing remyelination, was employed as targeting ligands and therapeutic drugs to functionalize the exosomes from mesenchymal stem cells. As a result, LJM-3064 aptamer-exosome conjugates promoted proliferation of OLN93 cells, suppressed inflammatory responses and reduced severity of multiple sclerosis in C57BL/6 mice model. However, applications of exosome drug delivery have been hindered by low yield and expensive preparation. Wan et al. proposed a more rapid and economic approach for possible clinical implementation. Researchers anchored cholesterol-poly-conjugated AS1411 aptamers onto living mouse dendritic cells membrane, followed by passing through micron-constrictions to generate exosome-mimic nanovesicles. They demonstrated that the aptamer-enhanced exosome-mimic nanovesicles effectively delivered paclitaxel to tumor cells both in vitro and in vivo [88].

## 3. Aptamer-Functionalized Non-Biological Materials for Therapeutic Applications

Aside from biological materials, the rapid development in biotechnology also has led to great advances in a variety of physical or chemical nano-sized materials for biomedical applications. Nowadays, the emergence of sophisticated synthetic techniques and characterization methodologies enables researchers to design and manufacture many non-biological materials with customized sizes, shapes, and dispersions. Their inherent features such as large surface area, ease of modification, high loading capacity, and enhanced permeability and retention (EPR) effect, consistently attract interest from researchers to explore their therapeutic potentials in drug delivery and therapeutic imaging [16,89,90]. Furthermore, some materials exhibit unique physiochemical properties such as surface plasma resonance (SPR) or magnetism [91,92]. To further enrich their therapeutic potential, the incorporation of targeting ligands such nanomaterials has been extensively investigated. Aptamers, due to their outstanding molecular recognition abilities, exhibit increasing popularity as targeting moieties on many non-biological materials, such as micelles, hydrogels, gold nanoparticles, silica nanoparticles, or liposomes. Below we review the recent advances in aptamer-functionalized nanomaterials and their potential applications in therapy.

### 3.1. Polymeric Nanomaterials

#### 3.1.1. Micelles

Micelles are polymeric materials that self-assemble as nanospheres by amphiphilic block copolymers via a thermodynamic process at a critical micelle concentration (CMC) [93]. They represent one effective delivery system especially for poorly water-soluble agents due to their hydrophobic reservoir cores. As nanocarriers, micelles hold advantages include strong biodegradability and biocompatibility, high drug payload, and prolonged circulation and retention time. To date, several promising micelle-based nanostructures including Paclitaxel-Micelles (NCT00912639, Phase IV) and Docetaxel-Micelles (NCT03585673, Phase II) have proceeded to clinical trials. To advance their applications, particularly in cancer therapy, researchers are modifying micelles with active targeting ligands such as aptamers.

The conjugation of aptamers with micelles was initiated by Mizuo Maeda’s group in 2007 [94]. Since then, a variety of aptamer-micelle conjugates have been developed. For therapeutic use, aptamers are typically incorporated at the surface of micelles for targeted drug delivery. Different chemical methods, such as base-pairing hybridization [95], 1-Ethyl-3-(3-dimethylaminopropyl)carbodiimide(EDC)-mediated conjugation [96], and click chemistry have been used to attach aptamers on micelle structures [97]. Using self-assembly, aptamers are usually densely packed on micelle nanostructures, creating multivalent effects which led to enhanced molecular recognition.

**Figure 4:**
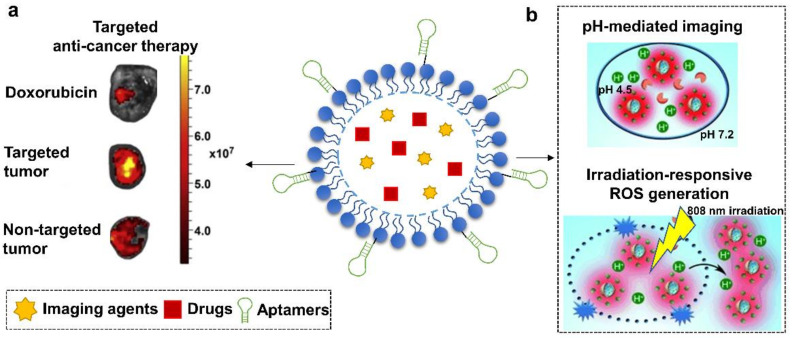
Schematic illustration of aptamer-functionalized micelles. They have been used to, (**a**) specifically deliver DOX to WR22Rν1 tumor-bearing mice for anti-prostate cancer therapy and (**b**) mediate pH/NIR-responsive breast cancer-specific imaging and therapy. (**a**) is modified with permission from reference [98]. Copyright 2013 Elsevier. (**b**) is adapted from reference [102]. Copyright 2014 Wiley Online Library.

In micelle-based nanostructures, aptamers mostly act as probes for cancer-associated markers such as prostate-specific membrane antigen (PSMA) [98], MUC1 [99], EGFR [100], human epidermal growth factor receptor 2 (HER2) [101], protein tyrosine kinase 7(PTK7) [29], and nucleolin [96]. In the study of Xu et al., aptamer-coated unimolecular micelles were assembled as spheres with diameter of 69 nm [98] (Figure 4a). Aptamers were selected against a prostate cancer biomarker, PSMA. In the aid of this nucleic acid probe, DOX-loaded micelles were able to specifically target PSMA-positive CWR22Rv1 cancer cells and demonstrated stronger anti-cancer cytotoxicity than aptamer-free treatments. Meanwhile, several cancer cell lines such as MDA-MB-231 cells [102] and Ramos cells [103] have also been found as direct targets for aptamers incorporated to micelles. Wu et al. in 2009 constructed a micelle nanostructure functionalized with aptamers specific to Ramos cells [103]. DNA aptamer TDO5 was synthesized with lipid tails which assembled into micelles at low CMC levels. The complex demonstrated enhanced binding and dynamic specificity to target cells in the mimic blood system, leading to efficient drug delivery to tumor sites. 

In addition to the selective recognition of targets, other benefits offered by aptamers for micelle-based nanocarriers include increased cellular uptake and reduced systematic toxicity. Li et al. in 2015 developed a micelle nanostructure that was composed of AS1411 aptamers, amphiphilic polymer Pluronic F127, and copolymers β-CD-PELA, for targeted drug delivery of DOX to human breast tumors [104]. Data revealed that aptamers AS1411 facilitated the targeted recognition of nucleolin-positive MCF-7 cells and increased the cellular uptake of DOX via nucleolin-mediated endocytosis. Meanwhile, DOX exhibited stronger anti-cancer efficacy but lower cardiotoxicity in mice bearing MCF-7 tumors than free drugs. Overall, the conjugation of AS1411 aptamers greatly strengthened the micelles-based system as drug nanocarriers.

Through combining multiple functional elements such as imaging agents or responsive stimuli, aptamers-micelles nanoconjugates achieved more versatile applications. For example, in the work carried out by Tian et al. in 2014, a multifunctional aptamer-micelle drug nanocarrier was constructed via integrating a pH-activatable fluorescent probe (BDP-668) and a near-infrared photosensitizer (R16FP) [102] (Figure 4b). Aptamers were functionalized to recognize target cancer cells, MDA-MB-231. The payload of R16FP was capable of mediating lysosomal destruction of target cells via generating reactive oxygen species (ROS) upon NIR irradiation. Meanwhile, the introduction of BDP-668 enabled the visualization of lysosomal pH change, thereby allowing to monitor the therapeutic progress in a real-time manner. This approach therefore might benefit cancer therapy through coupling precise drug delivery to imaging-guided therapy.

New examples are also found in peptide amphiphile micelles (PAMs)-associated research. PAMs are of particular interest as drug vehicles since they are designed to contain an intrinsic therapeutic peptide moiety, allowing a higher payload than other systems. Smith et al. attempted to conjugate aptamers to PAMs to construct targeted drug delivery systems for specific cancer therapy applications. Aptamers bound to human B-cell leukemia cells (C10.36) were attached to PAMs with anti-tail amphiphile via base pairing hybridization [95]. The system was proven to remain stable over 4 h in biofluids and exhibit specific cell targeting ability, leading to enhanced uptake by human B-cell leukemia cells. In addition, an extended study was reported from Samuel I. Stupp’s group, in which they coupled PDGF-BB-specific DNA aptamers to peptide amphiphile nanofibers (PANs) [17]. PAN is a material constructed by similar components of PAM, but assembles into an elongated nanofiber-like structure, which allows it to display high density of biological moieties on the surface. In Prof. Stupp’s research, PANs significantly empowered the PDGF-BB aptamers by enhancing their binding affinity and nuclease-resistant stability, leading to enhanced inhibition efficacy on PDGF-BB-induced proliferation of fibroblasts.

#### 3.1.2. Hydrogels

Hydrogels are made up of hydrophilic, cross-linked networks of polymers, al-lowing tissue-like elasticity and diffusivity of bioactive molecules. Studies have de-signed hydrogels to respond to various stimuli, including pH [105], temperature [106], light exposure [107], magnetic fields [108], and ionic strength [109]. Incorporation of aptamers into hydrogels have greatly expanded the range of applications of hydrogels in their use in biotechnology and biomedicine. Aptamers offer hydrogels many advantages. Aside from targeted delivery, other advantages include enhanced control of gel-sol transition, volume change, and molecule capture and release from hydrogel systems [110]. The following section reviews the applications of aptamer-integrated hydrogels for therapy.

**Figure 5:**
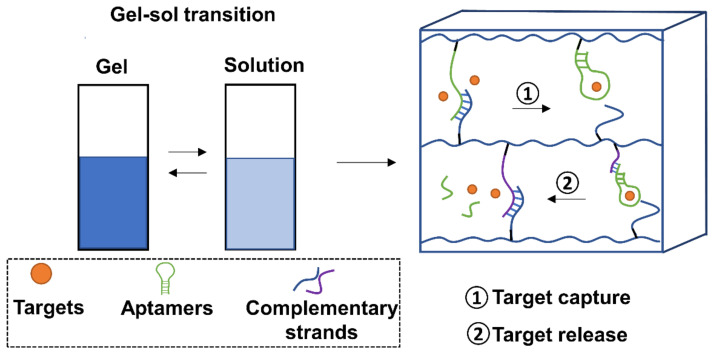
Schematic representation of the gel-sol transition of aptamer-decorated hydrogels. For target capture, aptamers in hydrogels would be de-hybridized from complementary strands to capture targets. For target release, aptamer-captured targets are released via a competitive hybridization from complementary oligonucleotides of aptamers or aptamer holders.

As drug carrier systems, hydrogels exhibit excellent characteristics such as high biocompatibility, low toxicity, and good swelling performance. One key challenge has been the ability to control the release and capture of drugs due to the material’s high permeability. Aptamers provide a strategy to overcome this limitation through the oligo-driven gel-sol transition (Figure 5).

In one of the earliest studies, Yang et al., developed a hydrogel cross-linked by adenosine-specific aptamers [77]. Upon adenosine binding, the gel-sol transition could be monitored using gold nanoparticles. This method could be adapted for use in the selective release of therapeutic agents. Following this, Wang et al. demonstrated that conjugation of aptamers within hydrogel systems could mediate the specific capture and release of single proteins, and even cells by utilizing cDNA hybridization schemes [111,112,113,114]. In notable work from the Wang group, an aptamer-functionalized hydrogel was able to achieve sustained release of B chain platelet-derived growth factor (PDGF-BB) by tuning analyte-ligand binding affinity [112]. Furthermore, the group used this system to develop a capture and release aptamer-mediated hydrogel for cancer cells [113,114]. In another notable study, drug delivery system by pulsating patterned release was proven possible. By adding partially complementary DNA strands, Soontorn-Worajit et al., designed a controllable aptamer-mediated PDGF-BB release system within hydrogels [115]. Their results showed sustained release of PDGF-BB for 6 days.

Many other examples of aptamer-mediated hydrogel capture and release systems are available. Wei et al., showed that their thrombin aptamer could capture targets in the gel state, then strand displacement led to the sol state and release of thrombin [116]. Liu et al., were able to design a step-release aptamer-functionalized hydrogel through sequential photoreaction [110]. Furthermore, Zhang et al. showed that the capture and controlled release of multiple proteins by aptamer-hydrogels was possible. The researchers used photoclick chemistry of complementary DNA strands to the aptamers [117]. Through this controlled DNA hybridization, they could successfully apply this system into human serum for “excellent cytocompatibility”. Sub-nanomolar levels of aptamer-functionalized hydrogels (termed nanogels) have also shown promise as drug release systems. Kang and colleagues designed a near-infrared (NIR) light-responsive aptamer drug release system for DOX [118]. Heating up the aptamer-nanogel with NIR rays led to dissociation of the aptamer-nanogel network. This resulted in the release of the entrapped DOX within cancer cells. 

More interesting examples include using aptamers-hydrogel systems to promote the attachment and growth of endothelial cells [119]. Future efforts might focus on exploring their possibilities for tissue remodeling and regeneration.

#### 3.1.3. Polymeric Nanoparticles

Polymeric nanoparticles are solid colloidal systems, which are gaining popularity because of their potential as drug delivery vehicles. They are mainly formed by synthetic hydrophilic polymers that include but are not limited to polyglycolide, polylactide (polylactic acid) (PLA) [120], polycaprolactone (PCL) [121], poly (lactic-co-glycolic acid) (PLGA) [122], polyethylene glycol (PEG) [123], and cationic polymers like polyethyleneimine (PEI) [124]. With different preparation processes, the structures of resultant polymeric nanoparticles vary from nanospheres to nanocapsules, allowing to load therapeutics throughout or inside the nanocarriers. The payloads in polymeric nano-particles can be released passively by erosion and diffusion, or actively in response to external triggers such as pH and heat [125]. 

**Figure 6:**
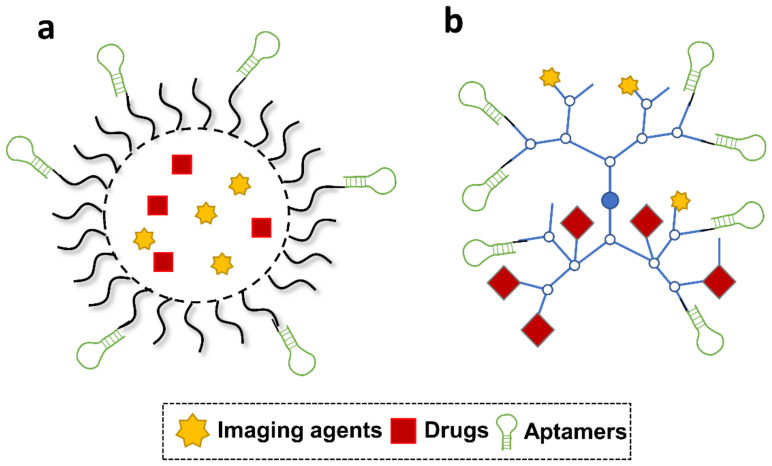
Schematic illustration of aptamer-functionalized polymeric nanoparticles and branched polymers. (**a**) Polymeric nanoparticles. (**b**) Branched polymeric nanostructures.

Due to the flexibility in chemical modification, aptamers have been attempted to functionalize polymeric nanoparticles for targeted cancer therapy. A generalized illustration of aptamer-decorated polymeric nanoparticles is shown in Figure 6a. As anchors at the surface, aptamers were found to be incorporated into different polymeric mate-rials, such as PLGA [126,127,128], PEI [129], PLA [130], poly(l-lysine) (PLL) [129], and poly(butylene adipate-co-terephthalate) (PBAT) [131]. Hence, drug nanocarriers benefit from enhanced cellular uptake, improved therapeutic efficacy, and reduced non-targeted cytotoxicity. In the work done by Chen et al, anti-PSMA aptamers were grafted on docetaxel (DTX)-loaded PLGA-b-PEG via EDC/NHS-mediated coupling chemistry [132]. In comparison with non-targeted nanocarriers, aptamer-functionalized systems, DTX-apt-NPs, showed enhanced anti-cancer effects and cellular uptake in LNCaP cells. The marked intracellular uptake was found to be associated with clathrin-dependent endocytosis. For targeted imaging-guided therapeutic applications, Aravind et al. constructed a versatile polymeric nanoparticle containing PLGA nano-particles, anti-nucleolin aptamers, chemotherapeutics paclitaxel (PTX), magnetic fluid, and fluorescent dye Nile Red (NR) [133]. Results demonstrated that cellular uptake of this nanoparticle could be well monitored via NR-derived optical imaging, facilitating a controllable and specific drug release in cancer therapy. High payload of nucleic acid drugs is another potential advantage of the polymeric nanoparticle system. PEI, as a cationic material, is well known to efficiently deliver nucleic acid drugs due to its positive charge. In Subramanian et al’s study, cationic PEI functionalized by anti-epithelial cell adhesion molecule (EpCAM) aptamers was designed to capture EpCAM siRNA drugs. The resulting system was proven to selectively downregulate EpCAM gene levels and inhibited the cell proliferation of two EpCAM+ cancer cell lines, namely MCF-7 and WERI-Rb1 cells, holding potentials for EpCAM+ cancer therapy [134].

#### 3.1.4. Branched Polymeric Nanostructures

Branched polymeric drug carriers such as dendrimers and hyperbranched polymers (HBP) have also attracted extensive attention due to their unique tree-like architecture. Dendrimers are composed of terminal units and dendritic units, whereas HBP consists of three structural domains including dendritic domain, linear domain, and terminal domain. They are, respectively, synthesized by divergent and polymerization approaches to generate nanostructures with unique structures, defined sizes, and diverse functional moieties at the surface, favoring further modification with aptamers.

As reported, aptamers have been incorporated into different branched polymers such as polyamidoamine (PAMAM) [135], hyperbranched poly(2-((2-(acryloyloxy)ethyl)disulfanyl)ethyl 4-cyano-4-(((propylthio)carbonothioyl)-thio)-pentanoate-co-poly(ethylene glycol) methacrylate) (HPAEG) [136], and Poly(PEGMA-co-TBMC-co-EDGMA-coCy5MA) (HBP-1) [137], for selective drug delivery. Figure 6b presents a typical design of ap-tamers-incorporated branched polymeric nanostructures. Among reported branched polymers, PAMAM is a cationic dendrimer used as nanocarriers particularly for nucleic acid drugs, which shows less biological toxicity, compared with PEI-based polymeric materials. In the work done by Xin Wu et al, RNA aptamer A10, specific to PSMA, was coupled to PAMAM by a polymeric spacer, PEG [135]. The constructed cargo finally achieved to deliver two microRNA drugs, miR-15a and miR-16-1, to targeting cells (LNCaP) for a specific anticancer therapy. Targeting genes of two microRNAs include BCL2, CCND1, and WNT3A were significantly suppressed with this specific treatment. In terms of HBP, they were found to be functionalized by aptamers against targets such as CCRF-CEM cells or 70 kilodalton heat shock proteins (HSP70s) [138,139]. In a poly-meric nanomedicine developed by Zhao et al., aptamers against HSP70s were covalently bound to Cy5-labelled HBP materials. When compared to free drugs, DOX loaded in the polymeric nanocomplex exhibited selective uptake and stronger regression for the breast solid tumors [140]. Meanwhile, because of the fluorescent Cy5 in the HBP, the behavior of the nanomedicine could be monitored, making the therapeutic process more controllable and evaluable.

Polymer hybrids have emerged as a new direction for developing polymeric nanocarriers. Hybridization can occur within typical polymers or between typical polymers and branched polymers, providing hybridized materials with additive advantages. Aptamer technology has extended possibilities for polymeric hybrids in drug delivery. For example, in a recent study conducted by Yang et al., poly (ethylene glycol) acrylate (PEGA) was conjugated to a photo-responsive branched polymer HBP, favoring the nanostructure with enhanced hydrophilicity, improved permeability, and prolonged circulation time. In addition, through incorporating dibenzocyclooctyne-modified sgc8 aptamers, this polymeric hybrid loaded with DOX achieved highly selective and controllable cytotoxicity against targeting CCRF-CEM cells [139]. Taken together, with more concept-to-proof studies, polymeric nanocarriers are promising to advance for clinical applications.

### 3.2. Inorganic Nanomaterials

#### 3.2.1. Gold Nanomaterials

Gold nanostructures have emerged as important biomedical tools due to advances in gold synthetic technology and their unique physiochemical properties [141]. Flexibility in synthesis allows customized gold materials with desirable sizes, shapes, and stabilities. Favorable characteristics in optics, electricity, magnetism, and biochemistry allow gold nanostructures to be attractive tools for bioimaging, drug delivery, radiation and photothermal therapy [142,143,144,145]. The ease of modification at the surface can endow gold materials with more therapeutic potential. In recent years, momentum is building to functionalize gold particles with aptamer ligands.

Aptamers offer notable advantages for therapeutics based on gold nanostructures, including enhanced drug efficacy and controllable drug release. Moreover, multifunctional aptamer-gold nanocomplexes can simultaneously achieve drug delivery, targeted therapy, and imaging. To date, dozens of aptamers-empowered gold nanoconjugates have been developed, mainly applied for anti-cancer therapy.

As targeting probes, aptamers can benefit gold nanocarrier-based treatments through enhanced targeting specificity, thereby leading to higher efficiency in bioimaging and photothermal therapy. Wu et al. in 2012 created Ag-Au nanostructures that incorporate the S2.2 aptamer, an aptamer selected against the cell surface cancer biomarker MUC1, which is highly expressed in MCF-7 cells of primary and metastatic breast cancers [146]. The nanostructures were able to target MCF-7 cells with high affinity and specificity. Meanwhile, because of the intrinsic surface-enhanced Ramon scattering (SERS) property, the aptamer-guided Ag-Au nanomaterial exhibited bioimaging potentials and showed excellent photothermal therapeutic potency against tumor cells in a specific manner.

In addition, aptamers with structure switching properties can control the release of loaded drugs, facilitating specific and controllable therapy based on gold nanostructures. For example, Wang et al. 2012 developed a targeted photodynamic therapy (PDT) and photothermal therapy (PTT) reagent by linking the photosensitizer molecule chlorin e6 (Ce6) to the surface of gold nanorods (AuNRs) via an aptamer switch probe (ASP) [147]. ASP changes conformation in the presence of the target cancer cells, driving Ce6 away from the gold surface that induces singlet oxygen for PDT upon light irradiation. Additionally, the absorption of radiation by the AuNRs enables further cell destruction via PTT. This multimodal PTT/PDT property of the AuNR-ASP-Ce6 conjugate results in a synergistic therapeutic effect.

Meanwhile, due to the intrinsic PTT effects of gold materials, synergistic anti-cancer therapy can be easily achieved in aptamer-gold nanomedicines by encapsulating a combined therapeutic agent. Yang et al. in 2015 synthesized a nanocomposite comprising of aptamer–gold nanoparticle-hybridized graphene oxide (Apt-AuNP-GO). This Apt-AuNP–GO nanocomposite was demonstrated for the targeted therapeutic response to tumor cells by NIR light-activatable photothermal therapy [148]. During NIR light-activatable PTT, heat shock proteins (HSPs) expression was modulated leading to a therapeutic response in cultured human breast cancer cells. Additionally, a combination therapy comprising of Apt-AuNP–GO NIR light-activatable photothermal therapy and an HSP70 inhibitor showed synergistic tumoricidal effects against cultured breast cancer cells.

**Figure 7:**
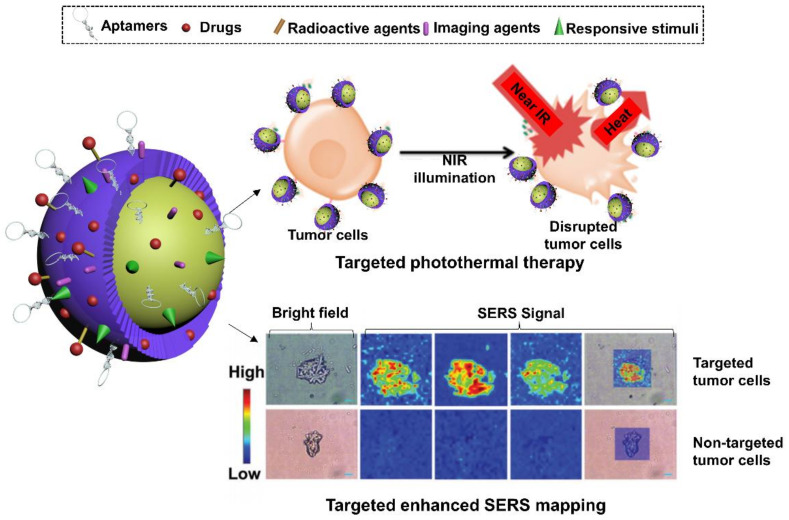
Schematic representation of aptamer-decorated gold nanostructures. They have been used to track A549-specifc tumors via gold-enhanced SERS imaging and kill tumor cells through NIR-triggered chemo-hyperthermia. It’s modified with permission from ref. [149]. Copyright 2019 RSC publishing.

Moreover, imaging-guided therapy has also been achieved recently in versatile aptamer-functionalized gold nanoconjugates. He et al. in 2019 fabricated A549 cell-targeting aptamer-labelled Raman tag-bridged gold nanoparticle (Au@Cu3(BT3)) NPs for synergistic chemo-photothermal therapy of tumors [149]. Raman signal agent, 4-MBA, was incorporated into the gold NPs. Aptamers and the chemotherapeutic DOX were modified on the surface of NPs for functionalization. After reaching the target cancer cells (A549) by the guide of aptamers, the release of the chemotherapeutic payload and hyperthermia generated from the Au NP core demonstrated synergistic effects in destroying cancer cells. Meanwhile, the Au NPs could also serve as SERS substrate to enhance the Raman signal of 4-MBA for cell imaging purposes, indicating the feasibility of traceable anti-cancer applications (Figure 7).

#### 3.2.2. Magnetic Nanoparticles

Magnetic nanoparticles are versatile biomedical tools that have broad applications in the clinic. Due to their intrinsic super-paramagnetism, magnetic nanoparticles have been applied for magnetic resonance imaging (MRI), drug delivery, hyperthermia cancer therapy, and the separation of specific cells [150,151,152,153]. Due to the remarkable molecular recognition and binding abilities, aptamers have been incorporated in many magnetic systems to achieve more specific molecular capture, delivery, and collection purposes.

In aptamer-functionalized magnetic composites, aptamers mostly serve as anchoring groups to specifically guide the nano-system to targeting tumor biomarkers or living cells, favoring the efficient delivery of different anti-cancer therapeutics. A few cases also use aptamers as handles to capture therapeutic agents for anti-bacteria applications. For example, in a study carried out by Dai et al., 2013, a magnetic core-plasmonic shell nanoparticle was constructed and functionalized by multidrug-resistant-bacteria (MDRB) antibodies [154]. Aptamer S8-7 was displayed on this nanocarrier to hold methylene blue for photodynamic killing and fluorescence imaging purposes. A combined photothermal destruction of targeting bacteria was also achieved by the coated gold. This system therefore holds potentials for multifunctional therapy of MDRB in clinical practice.

Given the majority of decorated aptamers act as targeting probes on magnetic drug vehicles, several representative examples of which will be highlighted in the following section.

**Figure 8:**
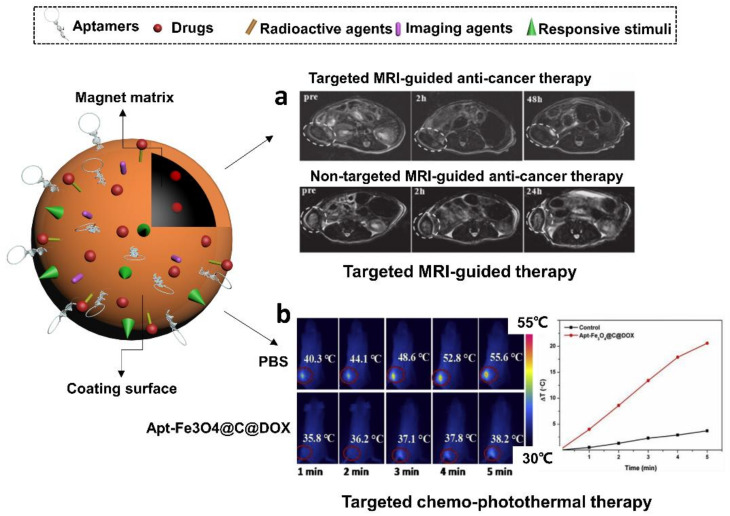
Schematic illustration of aptamer-functionalized magnetic nanomaterials. They have been used to (**a**) monitor the specific MRI-guided anti-prostate cancer therapy via DOX and (**b**) perform synergistic anti-cancer therapy mediated by DOX and hyperthermia in the guidance of MR imaging. (**a**) is adapted from reference [155]. Copyright 2011 Wiley Online Library. (**b**) is modified with permission of reference [157]. Copyright 2019 Elsevier.

As expected, the modification of aptamers enables magnetic nanocarriers to achieve targeted drug delivery and specific MR imaging. Yu et al. in 2011 conjugated PSMA-specific aptamers to thermally cross-linked superparamagnetic iron oxide nanoparticles to form a prostate cancer-specific nanotheranostic agent [155] (Figure 8a). Authors demonstrated that their prostate cancer-specific nanotheranostic agent was capable of simultaneous detection of target prostate tumors by MRI, delivery of the anti-cancer drug DOX to the targeted tumor sites and monitoring of the subsequent therapeutic responses of the tumors. In addition, Pilapong et al., 2014 conjugated an aptamer targeting EpCAM, to a polyvalent carboxymethyl cellulose modified magnetic nanoparticle (CMC-MNP) [156]. These EpCAM aptamer conjugated CMC-MNPs (Ep-MNPs) were demonstrated in the imaging application of in vitro MRI and targeted drug delivery to cells via Ep-MNP DOX loading.

Aside from that, aptamers with remarkable tumor-targeting capability are able to enhance the PTT efficiency of magnetic nanoparticles in cancer therapy. The encapsulation of other non-magnetic payloads could further strengthen these composite nanomedicines by synergistic therapeutic effects. For example, Aravind et al. in 2013 achieved targeted chemotherapy, sustained drug release and optical imaging using aptamer loaded magnetic fluid and paclitaxel loaded fluorescently labeled poly (D, L-lactide-co-glycolide) nanoparticles (Apt-MF-NR-PLGA NPs) [133]. The aptamer was used to conjugate nucleolin, a protein with elevated expression associated to worse cancer prognosis. Apart from the targeted delivery of paclitaxel via the nucleolin-specific aptamer, the magnetic polymer vehicles could also induce hyperthermia or magnetically guide the particles to tumor regions. In a recent study conducted by Zhao et al. in 2019., authors created aptamer-functionalized Fe_3_O_4_@carbon@DOX NPs (Apt-Fe_3_O_4_@C@DOX) and demonstrated their application in the synergetic chemophotothermal therapy (CPTT) [157] (Figure 8b). The sgc8 aptamer was used to functionalize the NPs to target the lung cancer model cell line A549. The Apt-Fe_3_O_4_@C@DOX NPs displayed both high photothermal conversion efficiency during PTT and pH/heat-induced DOX release. In vitro cytotoxicity assays indicated that the combined chemo-PTT approach shows greater toxicity toward lung adenocarcinoma cells (A549) than PTT or chemotherapy alone. Additionally, Apt-Fe_3_O_4_@C@DOX NPs presented decreasing contrast enhancement of MRI signals, meaning they have potential applications as contrast agents for MR imaging of tumor tissues.

#### 3.2.3. Quantum Dots

Quantum dots (QDs) are nanometer-sized semiconductor particles and present core-shell structures, which are composed of group II/VI elements such as CdSe and CdTe or group III/V elements such as InP and InAs [158,159]. They have gained extensive attention in recent years as fluorescent probes and energy mediators [160,161]. The inherent physicochemical properties endow QDs with impressive functions as robust fluorescent imaging agents and photosensitizer enhancers. Versatile QDs have been established by conjugating aptamer strands to achieve targeted therapeutic effects.

**Figure 9:**
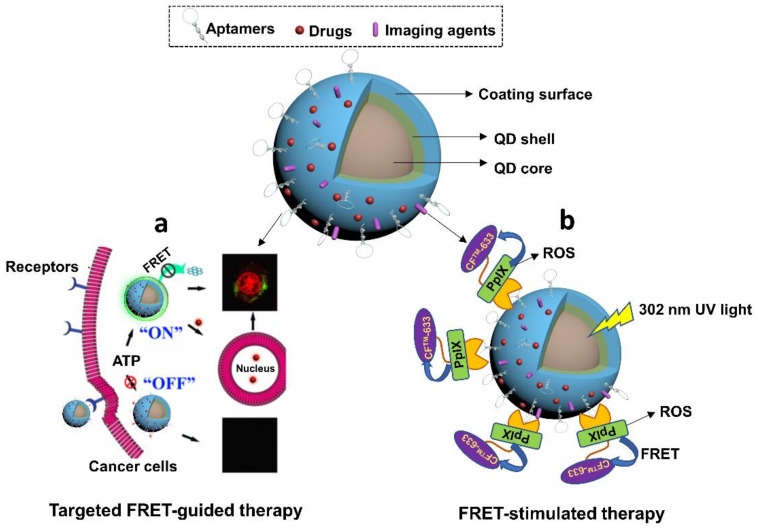
Schematic diagram of aptamer-empowered QDs nanomaterials. (**a**) ATP-triggered DOX release and FRET-guided targeted anti-cancer therapy. It’s modified with permission of reference [164]. Copyright 2015 ACS publishing. (**b**) UV-mediated FRET imaging and ROS-driven targeted photodynamic therapy of cervical cancer. It’s modified with permission of reference [165]. Copyright 2016 RSC publishing.

To date, aptamers-QDs conjugates have been mainly designed for cancer therapy and optical imaging. Aptamers normally attach to QDs via covalent bonds, and they could well maintain their targeting capabilities due to the minimal steric interference from excessively nano-sized QDs molecules. The first trial of immobilizing aptamers to QDs was found in the study of Zhang et al. in 2004 [162]. This group attempted to construct a QDs-based sensor and drug carrier for PSMA-overexpressed tumors via attaching PSMA-specific RNA aptamers. As suggested by recent studies, the incorporation of aptamers could benefit traditional QDs nanocarriers from increased imaging specificity, enhanced drug efficacy, reduced undesirable cytotoxicity and controllable release of loading agents. For instance, Savla et al., ever designed a DOX-loaded aptamers-QDs nanoconjugate for ovarian cancer therapy [163]. Aptamers were selectively against MUC1, a protein that’s overexpressed in a specific ovarian carcinoma cell line. Imaging data demonstrated that this nanomedicine preferentially accumulated in ovarian tumor and exerted stronger cytotoxicity than free drugs to target cells. In addition, in the study of Zheng et al., ATP-specific aptamers attached to graphene QDs (GQDs), were used to control the release of DOX and turn on the fluorescent signals quenched by GQDs in mesoporous silica nanoparticles, leading to real-time monitoring of drug release and efficient drug transport [164] (Figure 9a).

Aside from aptamers-derived advantages, QDs also provide several prominent benefits to aptamers-QDs treatments due to their intrinsic unique properties. One benefit is to empower photosensitizers in cancer therapy. Due to the FRET feature, QDs are able to mediate energy transfer to different photosensitizers and enrich their photodynamic therapeutic effects. In one research conducted by Singh et al., a nanoconjugate was constructed by coupling three modules, including ZnSe/ZnS QDs, MUC1 aptamers, and photosensitizer (protoporphyrin IX, PPIX) [165] (Figure 9b). Upon the specific binding to MUC1 peptides, the FRET between QDs and PPIX mediated QDs to activate PPIX for ROS generation for killing cancer cells. Meanwhile, the FRET between PPIX and CF^TM^ 633 amine dye (CF dye) triggered the fluorescent signals of CF to visualize the therapy process, implying the potential of using this system for specific programmable photodynamic cancer therapy. Another potential benefit from QDs in aptamer-functionalized nanomedicines is the synergistic anti-cancer effects. There are studies finding that some QDs exhibit inherent photothermal therapeutic effects upon exposure to NIR laser at 808 nm. This property has been used to design advanced anti-cancer treatments. For example, Cao et al., synthesized an aptamer-functionalized GQDs agent that was conjugated with PEGylated porphyrin derivatives (P) [166]. P, as a photosensitizer, was able to mediate photodynamic therapy. Moreover, GQDs exhibited abilities to ablate cancer cells under 808 nm laser via PTT effects. The integration of both therapeutics enabled a synergistic anti-cancer therapy for specific tumors.

Recent progress relating to aptamer-QD nanoconjugates has been significant for black phosphorus based QDs (BPQDs). In comparison with traditional QDs, BPQDs hold advantages such as outstanding photocatalysis activities in PDT, and broad photo-absorption from UV to NIR in PTT [167]. However, their anti-cancer therapeutic applications were hindered by low specificity and instability in the physiological environment [168]. To address his, Lan et al. attempted to functionalized BPQDs with hepatocellular carcinoma (HCC)-specific aptamers, TLS11a, along with a mesoporous silica framework (BMSF) [169]. Results revealed that the nano-catalytic system was able to perform an active targeting of HCC for programmable killing of cancer cells.

### 3.3. Silica Nanoparticles

Silica nanoparticles are mainstream ceramic materials, among which mesoporous silica nanoparticles (MSNs) are attracting increasing interest as drug carriers [170,171]. The development of synthetic technology allows us to design MSNs with large pore volumes, mesostructured sizes, great distribution area at surface, and high density of silanol groups, favoring their drug delivery applications particularly in high payload and the ease of conjugating functional groups [172,173,174,175]. Aptamers, as one type of specific targeting ligand, offer MSNs lots of benefits as drug carriers.

For therapeutic purposes, aptamer-MSNs conjugates mainly functionalize as drug nanocarriers, drug release modulators or in vivo drug trackers. Modification of aptamers benefits MSN-based nanocarriers through enhanced targeting precision. In the study of Gao et al., thrombin-binding aptamers (TBA) were tethered to the lipid-coated MSNs to construct a TBA-lipid-MSN nanocarrier of the chemotherapeutic drug, docetaxel (Dtxl), for anti-cancer therapy [135,176]. This bioconjugate was proven to strengthen anti-cancer effects of Dtxl via targeted drug delivery and release mediated by TBA in thrombin-overexpressing cancer cells holding potential as a potent specific suppressor of thrombin-positive tumors.

**Figure 10:**
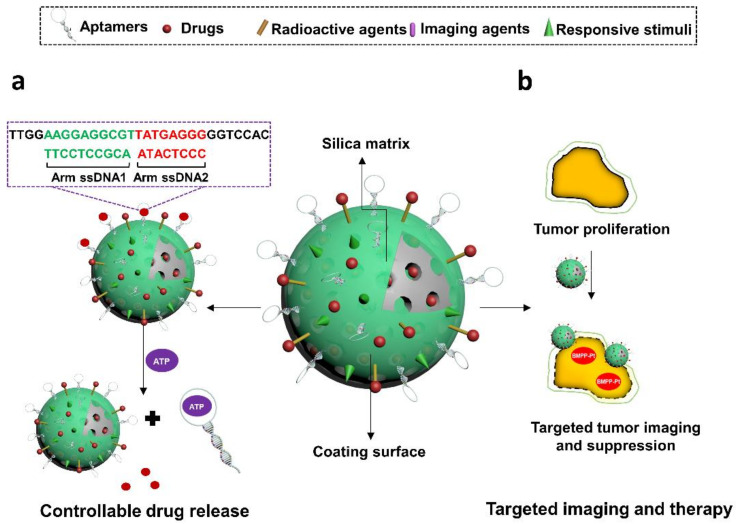
Illustration of aptamer-decorated silica nanomaterials. They have been used to, (**a**) control the release of Ru(bipy)^32+^ by de-hybridizing from complementary oligos in a ATP-responsive manner, and (**b**) guide BMPP-Pt to specifically image and suppress Huh7 cancer cells. (**a**) is modified with permission of reference [178]. Copyright 2012 ACS publishing. (**b**) is adapted from reference [186]. Copyright 2018 RSC publishing.

Controllable drug release is another benefit offered by aptamers to MSNs-based drug vehicles. In some studies, aptamers on MSNs were designed as efficient nanogates for delivered drugs due to their switchable structures upon binding to target cells or dedicated triggers [164,177]. For instance, He et al. in 2012 designed an ATP-responsive aptamers-decorated MSNs device [177] (Figure 10a). In this system, ATP-specific aptamers were initially hybridized with two single stranded DNA (ssDNA1 and ssDNA2), which were then grafted onto MSNs via click chemistry strategy, leading to the blockage of MSNs pores and no release of loading agents. While in the presence of ATP, aptamers were attracted by their targets and later detached from pores, resulting in the disruption of ssDNA1-ssDNA2-aptamer complex and release of guest molecules in MSNs. This concept could also be applied for designing other target-responsive MSNs systems via using aptamers as caps. Besides, other nanokeepers like light-sensitive graphene oxide [178], glutathione-responsive AgNPs [179,180], glutathione-responsive MnO2 [181], redox-responsive cytochrome C [182], or pH-sensitive hydrochloride dopamine [183], have also achieved expected effects for manipulating drug release in aptamer-MSN hybrids. Li et al. in 2017 constructed MSNs nanocarriers that were functionalized with EpCAM aptamers to deliver DM1 for targeted therapy of colorectal cancer [183]. EpCAM is an overexpressed surface biomarker in human colon adenocarcinomas. Decorated aptamers functionalized to guide the MSNs system to target cells and reduce systematic toxicity. Hydrochloride dopamine (PDA), a pH-sensitive agent, was coated on MSNs and acted as gatekeepers of DM1 in response to the pH stimulus in the tumor microenvironment. The resultant drug delivery device was found to exhibit enhanced cytotoxicity specific to an EpCAM-positive colon cancer cell line, SW480, and mitigated toxicity to normal cells.

Meanwhile, the flexibility of modifying aptamers or silica surface allows integration of more functional groups onto aptamers-MSN bioconjugates. Since real-time monitoring is an important concept of a smart drug delivery system, imaging technology has also been included in designing versatile aptamers-MSN nanocarriers. The signal materials coupled to the functional vehicle are diverse, including but not limited to NIR dyes, radionuclide chelation agents (eg. DOTA) [184], two-photon dyes [181], MRI contrast agents, or aggregation induced emission (AIE) compounds [185]. They are usually conjugated to aptamers or the surface of silicas or are encapsulated into the shell of MSNs. Upon the binding to specific triggers, signals can be well stimulated and monitored, which render the whole system with cell recognition or drug release tracking properties. In the study of Tang et al., authors synthesized versatile MSNs that were modified with positron emission tomography dye (DOTA-sil) and near-infrared dye (NIR-sil) for dual imaging of lymph node metastases [141]. Nucleolin-specific aptamers, AS1411, were incorporated to guide the system to targeted sites for real-time monitoring of the metastatic progress of tumors. This concept might facilitate the instant detection or intervention in cancer therapy. Besides that, Pasha et al. in 2018 designed an anti-EpCAM aptamer-functionalized MSNs system to encapsulate a dual functional compound, platinum-based “aggregation induced emission (AIE)” molecule (BMPP-Pt) [185] (Figure 10b). The component of BMPP-Pt is bis(diphenylphosphino)methane phenylpyridine platinum (II) chloride, holds potentials for theranostic applications in cancers by inducing cell apoptosis and emitting dose-dependent fluorescent signals. As benefited by the novel delivery system, BMPP-Pt was able to efficiently internalize and kill cancer cells, as well as perform intracellular fluorescent imaging, potentially extending its applications for cancer therapy. There is clear potential, but for clinical translation, challenges remain such as rapid filtration from kidney due to small sizes of both aptamers and MSNs, which will need to be overcome.

### 3.4. Carbon Materials

**Figure 11:**
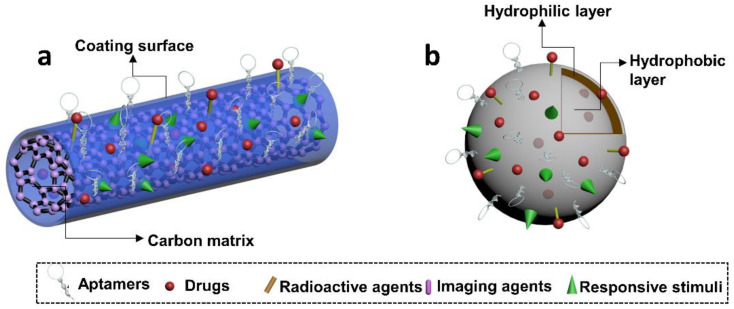
Schematic illustration of aptamer-functionalized carbon and liposome nanomaterials. (**a**,**b**) are aptamer-decorated versatile carbon and liposome nanomaterials, respectively.

One-dimensional nanostructures, in particular carbon nanotubes (CNTs), have seen increasing interest as not only sensitive biosensors, but also as drug delivery vehicles. CNTs are highly sensitive to the extra charges from adsorbed molecules on its surface, easily modified, and have a large surface area for drug loading, with high in vivo stability [186]. When coupled with an aptamer’s high affinity to bind directly to its target, the field of aptamer-CNT therapeutics has seen effective and rapid progress [187,188,189,190,191]. Thus, this review section will cover many of the current works in CNT–aptamer conjugates that have shown to enhance the efficiency of therapeutic delivery (Figure 11a).

In 2015, Mohammadi et al. showed that using single-walled carbon nanotubes (SWNT)-linked RNA aptamers could significantly enhance the treatment efficacy of cancer therapeutics [187]. To target EpCAM-positive solid tumors, the group synthesized an RNA aptamer against EpCAM which was attached to a SWNT conjugated to piperazine–polyethylenimine derivative. Ultimately, the aptamer-SWNT complex was able to be delivered into EpCAM positive tumor cells and induce apoptosis through silencing of BCL9I by piperazine–polyethylenimine.

Interestingly, SWNTs themselves have been shown to have antibacterial activity. The cobalt metal residues on SWNTs degraded bacterial cell walls causing cell death, leading to a suggestion that conjugation with aptamers may lead to “nano darts” against bacterial aggregation [188]. 

Another novel use of aptamer linked SWNTs is in photodynamic therapy (PDT). A complex of DNA aptamer, SWNTs and photosensitizer were designed for controllable single oxygen generation (SOG) [189]. In the absence of target α-thrombin, the close proximity of the photosensitizer to the SWNT surface causes quenching of SOG. In the presence of its target, the DNA aptamer detaches from the SWNT to bind to α-thrombin, resulting in SOG for PDT applications. Furthermore, the drug daunorubicin was linked with a leukemia-targeting aptamer-SWNT to enhance drug delivery into T-cells in a pH-dependent manner [192]. 

In a more recent study, aptamer-linked SWNTs were used to enable NIR laser-controlled cancer cell targeting and therapy. SWNTs were linked to sgc8-specific aptamers that were controlled using complementary DNA strands [190]. NIR laser exposure triggered DNA strands to dehybridize, exposing the aptamers so they can specifically recognize target sgc8, thereby guiding the delivery of DOX-loaded SWNTs.

Multi-walled carbon nanotubes (MWNTs) consist of multiple cylindrical layers of graphene. Although they are not as well-defined as SWNTs due to their structural complexity, they have been shown to provide several advantages over SWNTs. Some of these advantages are higher stability, and ease of modification [193]. In one of the first studies, in 2010 Bossche et al., constructed an aptamer linked MWNT that could easily translocate into the cytosol of different cell types [186]. Importantly this transportation method was found to be independent of receptor-mediated uptake, due in large part to the natural ability of CNTs to cross cellular membranes. 

There have also been significant advances in the field of three-dimensional carbon nanotubes (3DN-CNTs). A notable study by Gedi et al. reported the sensitive detection and imaging of ovarian cancer markers by aptamer-antibody pairing on 3DN-CNTs [191]. The 3DN-CNT was constructed on a chip surface and the aptamer-antibody pairing resulted in significantly higher surface loading density. They were able to achieve higher sensitivity and broader detection range compared to other fluorescent assays based on graphene oxide or traditional enzyme-linked immunosorbent assays [191].

### 3.5. Liposomes

Liposomes were first established in 1965 [194], and later developed as preferable drug delivery systems. They are typically structured as a spherical sac of phospholipids enclosing an aqueous core, which allows drugs to be encapsulated, either in the aqueous core, in the phospholipid bilayer, or at the interface layer. As drug carriers, liposomes apparently render biomedicines with significant advantages include strong biocompatibility and biodegradability, high drug loading efficiency, flexibility in fabrication, and ease of intracellular uptake. Such merits have brought many liposome-based delivery systems to clinical trials or even practical applications [195,196]. Emerging attempts are being made to develop aptamer-embedded liposomes, which would provide more potential for therapeutic applications. 

Intergradation between aptamers and liposomes can be achieved by either non-covalent or covalent coupling [197]. Non-covalent attachment, particularly electrostatic interaction, is usually used to incorporate negatively charged aptamers to cat-ionic liposomes. However, covalent attachment is typically preferred to generate ap-tamers-liposomes conjugates, even in cases with cationic liposomes. Often, covalent bonds carry advantages as they are relatively stronger and rarely affected by environment factors such as pH, temperature, or ionic strength. Studies have shown that aptamers can be either encapsulated into the aqueous core or attached at the surface of liposomes. Aptamers in the former case can serve as therapeutics. Liposomes benefit such treatments to achieve longer retention time, stronger biostability and enhanced cellular uptake. Aptamer drugs such as R12-2 (inhibit HIV virus infection) has been delivered in this way and exhibited superior efficacy than their free forms [198]. Aptamers can be encapsulated into cationic liposomes with their targeting drugs to enhance the drug-loading efficiency via optimizing charge and drug/aptamer ratio [199]. Aptamers are typically functionalized at the surface of liposomes (Figure 11b). In such scenario, most aptamers attach biomarkers, receptors, or cells/tissues involved in human cancers. Several common targets for aptamers are PTK7, E-selectin, CD44 antigen, PSMA, nucleolin, transferrin receptor (TfR), and EGFR [197]. Specific recognition significantly increases the intracellular uptake and cytotoxicity of liposome-based treatments, relative to their non-targeted counterparts. Recent developments in aptamer-modified liposomes have focused on rendering them with versatile functions. Specific delivery, real-time imaging, and programmable drug release were also achieved in latest aptamer-liposome systems through incorporating imaging agents such as quantum dots [200], or photothermal materials such as AuNSs [201]. To advance liposome delivery technology, researchers are modifying liposomes with polymeric materials such as PEGs [202]. Besides that, multiple-dose administration was suggested to maximize the cellular uptake of nanomedicines delivered by aptamer-based liposome systems [197].

### 3.6. Other Non-Biological Nanomaterials

Aptamers have also been found to functionalize nanomaterials such as metal-organic frameworks [203], upconversion nanoparticles [204], and acoustic droplets [205]. Metal-Organic frameworks (MOFs) are a class of porous coordination polymers that are constructed from metal containing nodes and organic nodes. The feature with tunable structure and function makes it feasible to integrate aptamers for high-performance applications in targeted biosensing and cancer therapy. MOFs could serve either as imaging probes or drug carriers due to their excellent optical, catalytical, and electrochemical properties. When functionalized with aptamers, MOFs were endowed with improved sensing or therapeutic efficiency. For example, in the study of Yuewu Zhao et al., nucleolin-specific AS1411 aptamers were utilized to modify ferric oxide-loaded MOFs, resulting in significant enrichment of MOFs at tumor sites to achieve improved therapeutic effects derived from PDT and chemo-dynamic therapy (CDT) [206]. Meanwhile, Hongmin Meng et al’s study demonstrated that the combination of G4-aptamer with Zr-MOFs led to specific recognition and efficient killing of cancer cells by the aptamer-attached photosensitizer, TMPyP4 [207].

Upconversion nanoparticles (UCNPs) are organic fluorophores and have attracted extensive attention since their recognition in the mid-1960s [208]. Properties of UCNPs such as small physical dimensions and biocompatibility enable facile coupling to ap-tamers. In recent years, aptamer-embedded UCNPs have been developed for various biomedical applications ranging from targeted bioimaging to cancer therapy. The re-search from Weijia Hou et al. reported a versatile aptamer-functionalized UCNP nano-system for cancer therapy [209]. The cancer cell-targeting sgc8 aptamer with photosensitizer, Ce6, was conjugated to UCNPs, which specifically guided the drug system to cancer cells. Upon activation by the NIR light, the cytotoxic skills from Ce6 were enhanced by the energy-transducer, UCNPs, leading to severe disruption of cancer cells.

In addition, researchers also attempted to attach aptamers to acoustic droplets. Acoustic droplets consist of perfluorocarbon that transit into gas bubbles when they become superheated by the ultrasound insonation, which could induce vascular occlusion and ultrasound ablation for cancer therapy [210]. To improve their targeting capabilities, fabrication of aptamers-incorporated acoustic droplets has been seen in Wang et al’s research. Herein, one DOX-loaded acoustic droplets-based nanocarrier was constructed. Sgc8 aptamers were conjugated on the surface and guided the system to target CCRF-CEM cells, leading to specific ultrasound imaging and therapy of CCRF-CEM-derived cancer [211].

## 4. Conclusions and Future Perspectives

In summary, recent advances in biotechnology and nanotechnology show significant potential of aptamers as nucleic acid carriers for biological drugs, or functional moieties in a variety of materials for advanced therapy or imaging applications. Such nucleic acid tools can augment therapeutic materials enabling guided delivery, ease of cellular uptake, gated drug release, and enhanced drug efficacy. Meanwhile, aptamer-based nanomaterials have also benefited from the rapid development of material science. Novel synthetic and characterization techniques allow the design of materials with optimized biological or physiochemical properties for a specific application. Table 1 lists some representative advantages and disadvantages of the therapeutic approaches reviewed in this paper.

For clinical translation, major obstacles encountered by these therapeutic strategies are discussed below. Firstly, one concern is the limited choice of aptamers for clinical use. Aptamers are short and single-stranded nucleic acids which are prone to degrade under the physiological environment containing nucleases. Meanwhile, most aptamers in literature were generated via in vitro selection. There is a high risk of losing their affinity in vivo in the physiological milieu. Therefore, aptamer stabilization methods and innovative SELEX strategies are urgently needed.

Secondly, the pharmacokinetics, pharmacodynamics, and off-target effects of aptamer-conjugated therapeutic materials are little understood in vivo. Relative to traditional materials-based nanomedicines, aptamer-functionalized systems are likely to acquire new features in size, structure, and surface charge, which may influence behavior in cellular uptake, biodistribution, metabolism and excretion in vivo. However, to date, only a few studies have assessed these therapeutic systems in vivo and differing results were found in different experimental models for the same nanocarriers [225]. Therefore, reliable and standardized animal models should be established to allow systematic and universal in vivo evaluations for aptamer-attached therapeutic candidates.

Thirdly, the biosafety issue of aptamers-based nanomedicines remains to be addressed prior to clinical trials. As foreign nucleic acids aptamers may hold some risks of genomic insertion, and immune responses need to be better understood. In addition, some materials functionalized by aptamers exhibit inherent cytotoxicity. For example, Cd-contained QDs have been found to cause DNA damage in cells and have high toxicity. To ensure biosafety for clinical trials, systematic toxicity evaluations of candidate aptamers-integrated treatments must be performed.

Regardless of the challenges, attempts are being made to address many of these issues. For example, for aptamers that are susceptible to nuclease degradation, researchers developed a variety of chemical modification and circularization approaches to enhance their serum stability, some of which significantly stabilized aptamers for long-term in vivo research [213,226,227]. Progress has also been seen in identifying robust aptamers with in vivo evolution strategies. Cheng et al., successfully selected RNA aptamers directly from mouse, which greatly reduced the risk of changing or losing functions when used in vivo [228]. In addition, more and more aptamer therapeutics such as NOX1257, Pegnivacogin, ARC1779, and SL1026 are undergoing pharmacokinetic, pharmacodynamic and biosafety studies, allowing better understanding of their in vivo behavior [229].

With the impressive advances in the construction and application of aptamer-conjugated materials systems, it is likely that aptamer-functionalized biomaterials will have a major beneficial impact on human health in future.

## Figures and Tables

**Table 1:** Advantages and disadvantages of aptamer-based therapeutics developed with different strategies.

Aptamer-Based Therapeutics	Functionalized Nanomaterials	Advantages	Disadvantages
Aptamer-enabled biological material system	Protein drugs [212]	1. Inherent drug efficacy 2. High payload capacity	1. Immune response 2. High production cost 3. Low blood-brain barrier permeability 4. Short shelf-life
	Nucleic acid drugs [213]	1. Low synthetic cost 2. Inherent drug efficacy 3. High payload capacity	1. Susceptibility to nuclease degradation 2. Rapid renal filtration 3. Risks of genetic mutations
	DNA nanostructures [214]	1. Programmed drug capture and release 2. High uptake 3. Ease of fabrication and modification	1. Susceptibility to nuclease degradation 2. Rapid renal filtration 3. Risks of genetic mutations
Aptamer-enabled non-biological material system	Micelles [215]	1. Ease of assembly 2. Prolonged circulation and retention time 3. Drugs to be protected from environmental stimuli, e.g., pH, enzymes, etc.	1. Limited payload capacity 2. Dependency of critical micelle concentration 3. Use only for lipophilic drugs
	Hydrogels [216]	1. Highly hydrophilic and biocompatible 2. Inherent tissue regenerative properties 3. Low cellular toxicity4. Relatively deformable to conform to the shape of implanted sites	1. Low tensile strength 2. Limited payload capacity 3. Limited drug homogeneity 4. Risks of drug burst-release
	Polymeric nanoparticles [217]	1. Controllable and sustained drug release 2. Flexible drug loading patterns 3. Multiple fabrication approaches 4. Tunable physiochemical properties	1. Difficulty to scale-up the Manufacturing 2. Insufficient research ontoxicological evaluations
	Branched polymeric Nanostructures [218]	1. Increased solubility of lipophilic drugs 2. High density of functional moieties 3. Fast cellular entry	1. High production cost 2. Cellular toxicity 3. Unsustainable drug release 4. Challenges for hydrophilic drugs
	Gold nanoparticles [219]	1. Ease of synthesis 2. Allow light-trigged drug release 3. Inherent photothermal anti-cancer effects 4. Low cellular toxicity 5. High payload capacity 6. Allow imaging-guided drug delivery	1. Difficulty for degradation and plasma clearance 2. Prone to aggregations 3. Undesirable accumulations at liver or spleen
	Magnetic nanoparticles [220]	1. High payload capacity 2. Allow MRI-guided drug delivery 3. Hyperthermia-mediated therapy 4. Controllable drug release	1. Highly magnet-dependent 2. Risks of causing vascular embolization 3. Undesirable accumulations at liver or spleen
	Quantum dots [221]	1. Fluorescence-guided drug delivery 2. Instinct anti-cancer effects	1. Rapid renal filtration 2. High cellular toxicity
	Silica nanoparticles [222]	1. High payload capacity 2. Tunable and uniform pore sizes	1. Only allow intravenous injection for administration 2. Low biodegradability
	Carbon materials [223]	1. High payload capacity 2. High cell membrane penetration capability 3. pH-mediated drug release	1. High hydrophobicity 2. High cytotoxicity
	Liposomes [224]	1. Low cytotoxicity 2. High cellular uptake 3. High biocompatibility 4. Drugs to be protected from environmental stimuli	1. Accelerated blood or reticuloendothelial system clearance 2. Low colloidal stability 3. High production cost

## Data Availability

Not applicable.
